# Advances in Gold Nanoparticle-Based Combined Cancer Therapy

**DOI:** 10.3390/nano10091671

**Published:** 2020-08-26

**Authors:** Kyle Bromma, Devika B. Chithrani

**Affiliations:** 1 Department of Physics and Astronomy, University of Victoria, Victoria, BC V8P 5C2, Canada; kbromma@uvic.ca; 2 British Columbia Cancer, Medical Physics, Victoria, BC V8R 6V5, Canada; 3 Centre for Advanced Materials and Related Technologies (CAMTEC), University of Victoria, Victoria, BC V8P 5C2, Canada; 4 Centre for Biomedical Research, University of Victoria, Victoria, BC V8P 5C2, Canada

**Keywords:** gold nanoparticles, radiation, chemotherapy, radiosensitizer, drug delivery system, chemoradiotherapy

## Abstract

According to the global cancer observatory (GLOBOCAN), there are approximately 18 million new cancer cases per year worldwide. Cancer therapies are largely limited to surgery, radiotherapy, and chemotherapy. In radiotherapy and chemotherapy, the maximum tolerated dose is presently being used to treat cancer patients. The integrated development of innovative nanoparticle (NP) based approaches will be a key to address one of the main issues in both radiotherapy and chemotherapy: normal tissue toxicity. Among other inorganic NP systems, gold nanoparticle (GNP) based systems offer the means to further improve chemotherapy through controlled delivery of chemotherapeutics, while local radiotherapy dose can be enhanced by targeting the GNPs to the tumor. There have been over 20 nanotechnology-based therapeutic products approved for clinical use in the past two decades. Hence, the goal of this review is to understand what we have achieved so far and what else we can do to accelerate clinical use of GNP-based therapeutic platforms to minimize normal tissue toxicity while increasing the efficacy of the treatment. Nanomedicine will revolutionize future cancer treatment options and our ultimate goal should be to develop treatments that have minimum side effects, for improving the quality of life of all cancer patients.

## 1. Introduction

According to American Cancer Society statistics in 2020, there will be an estimated 1.8 million new cancer cases diagnosed and 606,520 cancer deaths in the United States alone. Cancer is an abnormal growth of cells caused by multiple changes in gene expression leading to deregulation of the balance of cell death and proliferation, ultimately leading to an evolving population of cells that can invade tissues and metastasize to other sites [1]. The main types of cancer treatments include surgery, chemotherapy and radiotherapy according to the Canadian Cancer Society [2]. The treatment plan of each cancer patient will vary depending on the type of cancer and the advancement of cancer [2,3]. Radiotherapy is one of the most widely used treatment approaches, being used in approximately 50% of all cancer patients. In radiotherapy, a high dose of ionizing radiation is delivered to the tumor site, which interacts with and excites the atoms inside the cancer cells, causing damage to important structures, ultimately killing the cell [4]. Currently, the clinic mainly employs gamma or X-ray photons, ion-based electrons, or protons as radiation sources in the treatment [5,6]. While radiotherapy is widely used in many different types of cancers, a major issue still present is the normal tissue toxicity [7]. A photon beam will irradiate some of the surrounding healthy tissue no matter how well shaped or conformed the beam is to the dimensions of the tumor, and this dose to normal tissue limits the amount of radiation a patient can receive [8].

Chemotherapy is also used to eradicate micro-metastases and to improve local control of the primary tumor [9]. In chemotherapy, anticancer drugs are administered either orally or intravenously to disrupt the rapid overgrowth of malignant cells [10,11]. Similar to radiotherapy, the side effects caused by anti-cancer drugs remain as one of the important limitations in the advancement of cancer treatment [12,13]. Therefore, we need to improve the bioavailability of the drug in the tumor region, while confining them to this target, to reduce the amount of the drug needed, and thus the number, and severity, of side effects [14]. Some nanoparticle (NP)-based therapeutic systems have already been introduced into the pharmaceutical market. For example, Doxil, a polyethylene glycol (PEG)-liposome containing Doxorubicin, is approved for AIDS-related Kaposi’s sarcoma, ovarian cancer, and multiple myeloma [15,16]. Liposomal drugs and polymer drug conjugates account for most of the FDA (Food and Drug Administration, Tulsa, OK, USA)-approved systems so far [17]. However, in radiotherapy, NP-driven radiosensitization strategies that use inorganic high-Z (atomic number) materials have been pursued to improve the local radiation dose and minimize the damage to surrounding healthy tissue [18]. The interaction of high-Z materials with therapeutic X-ray photons results in an increase in the production of cell damaging species, such as free radicals and low energy electrons [19,20]. Inorganic NP systems such as gold nanoparticles (GNPs), silver NPs, gadolinium-based NPs, lanthanide-based NPs, and titanium oxide nanotubes have been reported as radiosensitizers [21,22,23,24,25,26,27]. Gadolinium-based NPs offer an innovative approach because of their capacity to act as a radiosensitizer as well as a powerful contrast agent in magnetic resonance imaging [26]. The high Z-nature of silver-based NPs along with their antimicrobial properties made them a good candidate in radiotherapy [27]. However, GNPs are the most widely used NP system in radiotherapy due to their ease of production, high Z-nature, advantageous surface chemistry, and biocompatibility [25,28,29,30].

There are different gold-based nanotherapeutic systems available, such as spherical GNPs, gold nanorods, gold nanoshells, gold nanoclusters, and GNP-incorporated liposomal nanoparticles, with many new anisotropic geometries being developed regularly. Spherical GNPs are the most commonly used gold-based nanotherapeutic, as their production is relatively simple and alteration of size and surface chemistry, such as conjugation with polyethylene glycol, is easily achieved [31,32]. Further, GNPs are heavily studied for use in the treatment of cancer through X-ray irradiation and as an anticancer drug carrier [33]. The use of gold nano-rods and gold nanoshells for the treatment of cancer involves the induction of hyperthermia, due to their larger cross-section at near-infrared (NIR) frequencies [34,35]. A comprehensive review of the use of gold-based nanomaterials such as gold nanoshells and gold nanorods in photothermal therapy has been described previously by Vines et al. [36]. It has also recently been shown that gold-based nanotherapeutics can absorb radiofrequency (RF) frequencies and generate heat, opening an avenue to treat more deep-set tumors with the use of gold and hyperthermia-based options [37]. Although more research must be completed, the use of RF waves with gold nanomaterials is very promising. Furthermore, due to the surface plasmon resonance effect present in GNPs, visible light irradiation can also allow for hyperthermia via photothermal therapy, recently shown by Mendes et al. with a green laser light in combination with 14 nm GNPs and doxorubicin [38]. However, the penetration depth of green light is even less than NIR and is thus limited in applicability [39]. Due to their theranostic benefits, such as imaging and biosensing, along with therapeutic properties such as drug delivery, gold nanoclusters have emerged as a useful tool [40,41]. The use of gold nanoclusters can allow for molecular imaging, improving diagnostics and imaging in the future [42]. Ultrasmall gold nanoclusters have also emerged as a useful technology due to their near 100% renal clearance, allowing for the improved probing of disease when utilized as a biosensor [43]. Lipid-based nanoparticles are an avenue that is being explored due to their ability to encapsulate GNPs for radiosensitization purposes and simultaneously act as a drug delivery platform [44]. Utilizing liposomal nanoparticles as a ‘smart’ drug carrier can allow for controlled release of the internalized cargo, such as in response a NIR light source, allowing more control over the treatment process [45].

GNP-based platforms are being researched and have been tested extensively in the field of cancer nanomedicine [46]. For example, a novel nanomedicine that conjugated human tumor necrosis factor alpha (rhTNF) and thiolated PEG onto the surface of colloidal GNPs (named CYT-6091) has been tested in phase 1 clinical trial in cancer patients [47]. The results from the CYT-6091 trial showed that doses up to 600 µg/g of rhTNF were administered without encountering dose-limiting toxicity and was less toxic than a treatment with just rhTNF, as evidenced by a lack of hypertension in patients. Furthermore, the GNPs had gathered in the tumor and mostly avoided healthy tissue. Other phase 1 clinical trials involved the use of PEGylated gold nanoshells around a silica nanoparticle, called AuroLase^®^, in head and neck, lung, and prostate cancer, with laser irradiation [48,49,50]. Results have, however, not translated to an effective treatment outcome. Another early phase 1 clinical trial involves the use of NU-0129, a platform consisting of nucleic acids attached to the surface of spherical GNPs [51]. The goal of this study is to use the conjugated nucleic acids to bypass the blood-brain barrier and target the BcL2L12 gene present in recurrent glioblastoma. If successful, this platform could supress this gene, which would lead to reduced proliferation and containing the spread of the tumor. However, translation of GNPs to the clinic is still in progress, and further optimization of protocols will have to be elucidated before the majority of research can move out of the preclinical stage, as described in the extensive review by Schuemann et al. [52].

**Figure 1:**
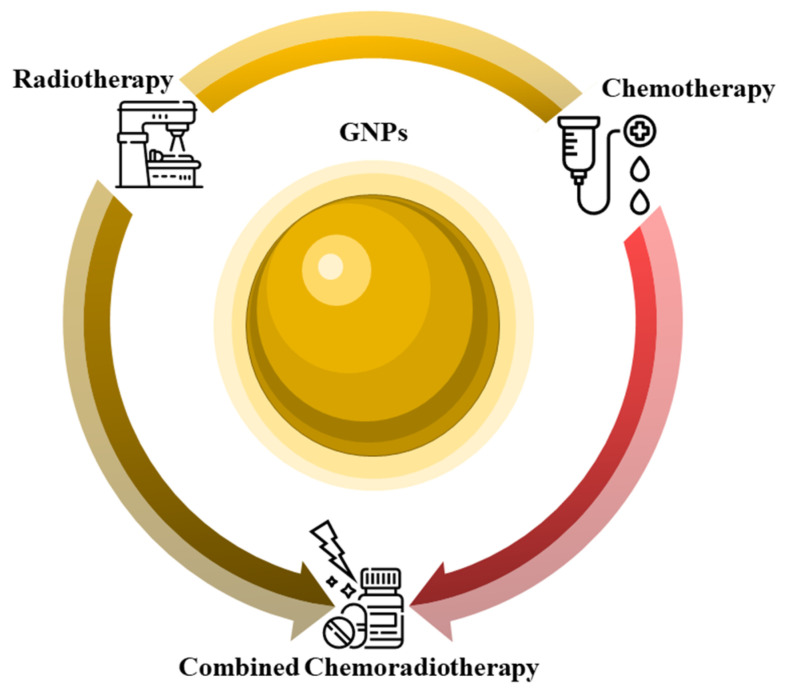
**Gold nanoparticle-based cancer therapeutics.** Radiotherapy and chemotherapy are the two main modalities, besides surgery, in treating cancer. However, normal tissue toxicity in both methods remains a large issue in limiting the effective dose to the tumor. Thus, gold nanomaterials have been introduced to improve the locally deposited dose into tumors and act as a drug delivery system. The combination of radiotherapy and chemotherapy, called chemoradiotherapy, allows for an optimum platform for eradicating the tumor and improving cancer therapeutics.

For patients with locally advanced disease, a combination of treatments, such as surgery with chemotherapy and/or radiotherapy is being used. A combination of chemotherapy and radiotherapy (referred to as chemoradiation) is a logical and reasonable approach that has greatly improved the cure rates of solid tumor [8,53]. This combined treatment modality provides local control of the primary tumor mass through radiation while tumor metastasis is suppressed through anticancer drugs [8]. One of the major limitations of chemoradiation as a treatment option is the normal-tissue toxicity, as either radiotherapy or chemotherapy can cause major normal tissue toxicity, as described previously. In order to overcome the normal tissue toxicity in current cancer treatment modalities mentioned previously, NPs are being used to enhance either the local radiation dose or improve delivery of anticancer drugs, or both, as seen in Figure 1. GNPs are one of the materials extensively tested for both radiotherapy and chemotherapy. Therefore, this review article will be focused on prospects of GNP-mediated cancer therapeutics.

Due to the large amount of recent interest in GNPs as a therapeutic agent, there have been many reviews on the topic [33,36,37,52,54,55,56,57,58,59,60,61]. Beik et al. have a recent, extensive review on the use of GNPs in various different modalities, including radiotherapy and chemotherapy, with a larger focus on photothermal therapy and combined treatment options [56]. However, the focus on radiotherapy is limited mainly to kV energy ranges, where GNPs have the largest differential in absorption cross section compared to soft tissue. To be clinically relevant in a larger variety of cancers, the efficacy of GNPs at an MV energy range needs to be explored. As previously mentioned, recent reviews on the use of irradiation in the NIR and RF range with gold nanomaterials for hyperthermia have shown promise [36,37]. Despite continuing research, however, irradiation involving X-rays dominate clinical treatment schemes, occurring in greater than 50% of patients [62]. Of all the gold nano-based therapeutics, spherical GNPs are extensively tested for both radiotherapy and chemotherapy. Therefore, this review article will be focused on prospects of GNP-mediated cancer therapeutics with clinically relevant radiotherapy, chemotherapy, and with a combined modality. This includes information that is necessary in order to improve efficacy, such as an understanding of GNP uptake at a cellular level, and how the size, shape, and functionalization of the GNPs alters effectiveness. In order to better understand the application of GNPs in cancer treatment, an introductory section is presented to understand the behavior of GNPs at a single cell level.

## 2. Intracellular Fate of Gold Nanoparticles Based on Their Physicochemical Properties

**Figure 2:**
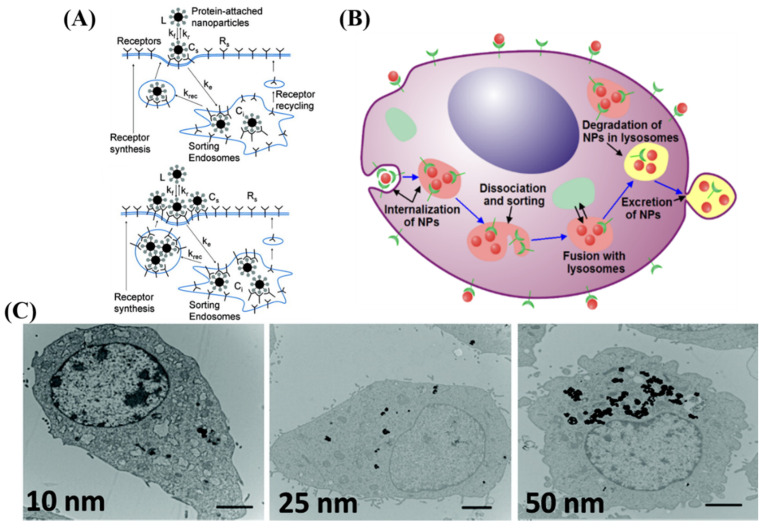
**Uptake of GNP by receptor-mediated endocytosis.** (**A**,**B**) Schematic illustrating pathway of citrate-capped GNP uptake into the cell. (**A**) Describes the entry and sort mechanism for a single NP and multiple NPs, while (**B**) describes the entire flow of internalization and excretion. Once GNPs are attached to the receptors on the surface of the cell, membrane invagination occurs followed by budding into the cell, forming a vesicle. The internalized GNPs are sorted inside the vesicle and eventually fuse with lysosomes. GNPs are then excreted out of the cell. (**C**) Transmission electron microscope images of rat kidney cells treated with three different sizes of GNPs. Scale bar is 2 μm. Reproduced with permission [68,70]. Copyright American Chemical Society, 2009, 2011.

There are different methods of entry into cells for NPs, including clathrin-mediated endocytosis, clathrin-caveolin independent endocytosis, and caveolae-mediated endocytosis [63]. Most NPs, including GNPs, enter the cell mostly via clathrin-mediated, or receptor-mediated, endocytosis (RME) [46,64,65,66,67,68]. The efficiency of the RME process depends on the interaction between molecules on the NP surface (ligands) and the cell membrane receptors. As illustrated in Figure 2A by Jin et al., cell surface receptors bind to molecules on surface of NPs, causing membrane wrapping of the NP with a corresponding increase in elastic energy [64,68]. The receptor-ligand binding immobilizes receptors causing configurational entropy to be reduced. More receptors diffuse to the wrapping site, driven by the local reduction in free energy, allowing the membrane to wrap completely around the particle [69].

RME is therefore an energy dependent process where the path of the NPs within the cell is explained in Figure 2B. NPs first reach the cell membrane and connect with the cell membrane receptors, which are mobile on the surface. Internalization of NPs occurs via invagination of the membrane, which then get trapped in endosomal vesicles. These internalized NPs are sorted inside the vesicle and eventually fuse with lysosomes, which can be seen within the cell as shown in Figure 2C by Ma et al. [70]. NPs are then excreted out of the cell. This intracellular path of NPs was further confirmed by Liu et al. by using a NP complex tagged with a fluorophore [71]. This group suggested that NPs are eventually transported to lysosomes by observing the co-localization of the fluorescently tagged NPs and lysosomes stained with lysotrackers.

**Figure 3:**
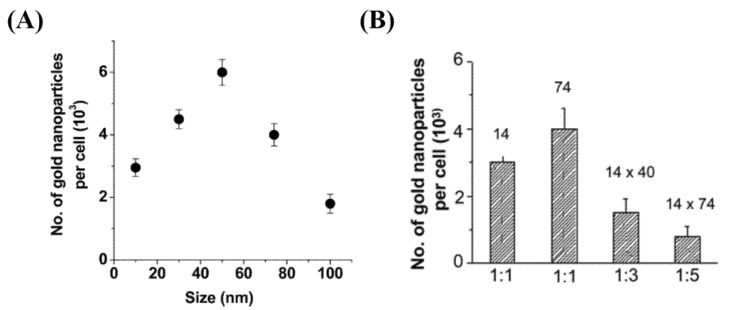
**Effect of size and shape on cellular uptake of gold nanoparticles.** (**A**) Dependence of gold nanoparticle cellular uptake as a funtion of their diameter. (**B**) Comparison of uptake of rod-shaped nanoparticles (aspect ratios 1:3 and 1:5) and spherical nanoparticles (1:1). Reproduced with permission from [66]. Copyright American Chemical Society, 2006.

The RME is also dependent on the size, shape, and surface properties of NPs. Chithrani et al. investigated the effect of both size and shape on GNP internalization (see Figure 3A,B) [66]. Among the size range of 10–100 nm, bare GNPs of diameter 50 nm had the highest uptake. They also found that the cellular uptake of rod-shaped NPs was lower than their spherical counter parts. This outcome was explained as a result of balance between energy needed for membrane wrapping of NPs and kinetics of receptor diffusion along the cell membrane [67,68]. They used citrate-capped NPs for the study which were not functionalized, where the RME process of the NPs was facilitated via non-specific binding of serum proteins on the NP surface once they were introduced to the tissue culture media [72]. However, it is important to optimize NPs properly for efficient in vivo delivery to the tumor.

There are many factors to consider when optimizing GNPs for use in an in vivo environment. For example, the administration route of the GNPs affects their absorption, toxicity, and tissue distribution [73,74]. Oral and intraperitoneal routes of administration had the largest toxicity, while a tail vein injection had the least, suggesting that an intravenous injection is most promising. Upon administration, the pharmacokinetics of the GNPs is another factor that must be optimized. GNPs exhibit very complex and varying pharmacokinetics, due the vast number of options in size, shape, and functionalization. Avoidance of opsonization and the reticuloendothelial system (e.g., liver and spleen), while also targeting the tumor, are important goals in nanotechnology [75].

**Figure 4:**
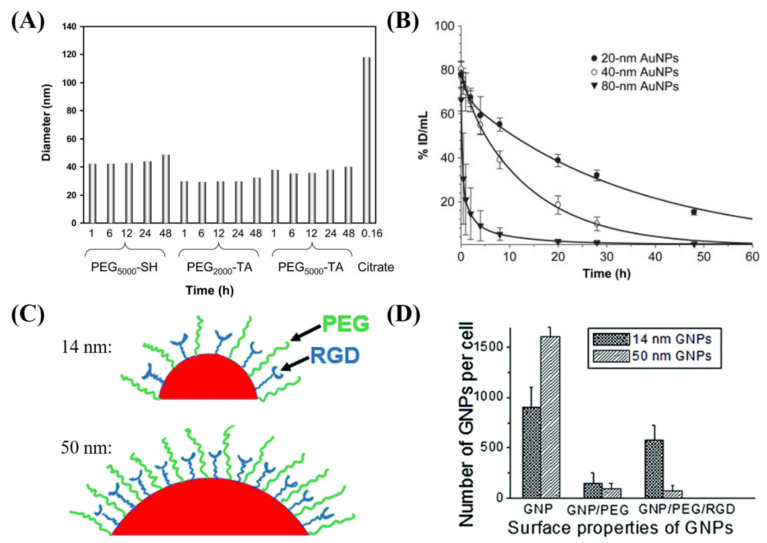
**Effect of functionalization on cellular uptake of gold nanoparticles.** (**A**) Diameter as measured using dynamic light scattering of GNPs functionalized with different PEG moieties, compared to bare GNPs. (**B**) Pharmacokinetics of different sized PEGylated GNPs expressed as a percentage of injected dose per gram of tissue in mice. (**C**) GNPs can be functionalized with PEG for stability and a peptide containing integrin binding domain RGD for targeting. (**D**) The use of the RGD functionalized GNPs allowed for improved uptake into tumors cells compared to GNPs functionalized with solely PEG. Reproduced with permission from [76,82]. Copyright Elsevier, 2009; Copyright Royal Society of Chemistry, 2015.

Prolonged in vivo residency time and preferential localization in tumors are key features of an efficient NP system [76]. If not functionalized properly, the opsonin protein in the blood plasma will attach to the NP surface, leading to the removal of the NP from the circulatory system by macrophages [77,78]. Furthermore, the protein corona that can form from interactions of the GNPs with blood, as a result of size, shape, charge, and functionalization, can alter the behavior of the nanoplatform [79]. Therefore, surface modifications of GNPs are performed to protect the particle from the environment and to target the particle to a specific cell or tissue type. This is critical, because the GNPs need to be present long enough for the process of accumulation within a tumor through its leaky vasculature, known as the enhanced permeability and retention (EPR) effect [80]. Previous studies have shown that the addition of PEG molecules to the surface of NPs increases blood circulation time [80,81,82]. The process of PEGylation allows for the ethylene glycol to form associations with water molecules, allowing for the formation of a protective hydrating layer, which in turn hinders protein adsorption and clearance by macrophages [83]. The stability of GNPs functionalized with PEG molecules was done by Zhang et al. in Figure 4A, who showed that PEGylated GNPs maintain stability over time, compared to bare GNPs who aggregate quickly [82]. GNPs functionalized with PEG molecules have also shown the capacity to evade the immune system and remain in the blood undetected by macrophages [76]. Further, Zhang et al. showed that GNPs maintained a large blood concentration over time for 20 nm and 40 nm PEGylated GNPs, as seen in Figure 4B [82]. However, the drawback of PEGylating the NP surface is that RME is very much retarded. To overcome this lower uptake of NPs, researchers have added targeting moieties to overcome the reduced NP uptake. One approach was to add a peptide containing arginine-glycine-aspartic acid (RGD) sequence, as performed by Cruje et al. in Figure 4C [77]. The RGD sequence can recognize the integrin αvβ3 that is highly expressed by several solid tumors and has demonstrably higher uptake than GNPs functionalized with just PEG [76,84]. Depending on the size of the PEG molecule and GNP, the uptake dynamics shown in Figure 4D was changed. For example, it was shown that smaller GNPs had a higher uptake compared to GNPs of diameter 50 nm [76,85]. The peptide and PEG molecules were on the order of 2 kDa and smaller NPs were able to maximize the ligand–receptor interaction of RGD peptide using their higher surface curvature [76,85].

Various factors can affect the pharmacokinetics of the GNPs. Depending on the size, the GNPs will have a different fate in vivo [86]. Smaller PEGylated GNPs of sizes 4 nm and 13 nm had high blood levels for 24 h and were cleared after 7 days, while larger GNPs (100 nm) were completed cleared after 24 h. Furthermore, the accumulation of smaller GNPs in the liver and spleen was peaked after 7 days, and in the mesenteric lymph node after a month, followed by clearance after 6 months. Larger GNPs were taken up into the liver, spleen, and mesenteric lymph node within 30 min. In general, larger GNPs concentrate in the kidney and spleen, and smaller GNPs are found throughout more organs [87]. Ultrasmall GNPs (<10 nm) have been studied due to their improved capabilities to be cleared from the reticuloendothelial system [88]. Further, Bugno et al. showed that that smaller GNPs (2 nm) have a three-fold increased tumor penetration compared to their larger counterparts (4 nm) [89]. As hypoxic regions far from capillaries tend to be the driver for treatment resistance, the ability to reach these regions with GNPs to increase local damage is a very important goal [90]. However, due to the large surface of curvature, despite surface coating with moieties like PEG, ultrasmall GNPs can have gaps that can be filled with blood proteins such as fibrinogen. As a result, smaller GNPs can contribute to an inflammatory response, due to their interactions with these proteins, highlighting the necessity for proper functionalization [91]. Another factor that impacts biodistribution is the surface charge, which can be controlled by various surface conjugations, such as with PEG [92,93]. The addition of PEG to 20 nm glucose-functionalized GNPs has been shown to increase the half-life period from 1.23 h to 6.17 h [94]. Furthermore, Geng et al. found that the functionalization of the GNPs lead to 20 times higher concentration in tumor tissue compared to normal tissue in the same organ, leading to an increase in damage to tumor following radiation [94]. This highlights the importance of proper functionalization to properly target GNPs to the tumor.

Other functionalization methods have also been tested to effectively target GNPs to tumors. In a variety of different epithelial cancers, epidermal growth factor receptors (EGFRs) can have significantly higher expression on cancer cells compared to normal cells [95]. Cetuximab (C225) is an antibody that allows for EGFR targeting, and has been shown to be effective at improving uptake compared to PEGylated GNPs in-vitro and in-vivo, by Kao et al. [96]. Another method involves the use of aptamer-based targeting. Aptamers are short single-stranded DNA or RNA oligonucleotides that are capable of binding to biological targets [97]. Aptamer-based GNPs can allow for specific targeting as well as aid in diagnostics [98]. Transferrin is a serum glycoprotein that can also be used to target GNPs to tumor cells, as there is an upregulation of receptors on metastatic and drug-resistant malignant cells [99]. The use of transferrin coated GNPs have been shown to improved uptake and allow for specific targeting to improve delivery of therapeutic agents [100]. Folic acid is another targeting molecule that can be employed, as the folate-receptor can be upregulated on human tumors while being minimally expressed on most normal tissue, as evidenced by Zhang et al. [99,101]. While there are many different functionalization modalities that can be employed, it is very important to test the efficiency of functionalized NP systems by varying their size, shape, and surface properties to optimize their internalization within tumor cells to cause the maximum damage. No matter what system that is employed, careful consideration of the functionalized GNPs with the protein corona that can form in vivo can allow for proper targeting and a predictable fate. [102] GNPs have also been associated with anti-inflammatory responses [103]. Thus, the toxicity of GNPs is an important factor that has been explored.

A number of groups studying GNP cytotoxicity concluded that GNP biocompatibility depends on size, surface properties and concentration [104,105]. Many experimental works reported that GNPs are non-toxic. For example, Connor et al. found various sizes (4, 12, 18 nm) and capping agents (citrate, cysteine, glucose, biotin, and cetyltrimethyl ammonium bromide) were nontoxic to K562 human leukemia cell line up to micromolar concentrations based on MTT assays [105]. Steckiewicz et al. found that the shape and concentration of the GNP complexes impact toxicity, with spheroidal GNPs (14 nm) imparting the least toxicity [106]. Sukla et al. observed lysine capped 35 nm GNPs did not show detectable cytotoxicity up to 100 μM concentration in RAW265.7 macrophage cells based on MTT assays [104]. It has been shown that PEGylated 12.1 nm sized GNPs incubated in HeLa cells had an IC_50_ of 0.477 mM [107]. Despite the many reports on non-toxicity of GNPs, contradictory research results are also present [108,109]. The lack of general consensus on NP toxicity is due to different experimental methods employed, incubation conditions (concentrations and exposure time), variability of sizes and functionalities of GNPs, variability of cell lines, and different measures and assays for toxicity [108,110]. However, most current research platforms are working in conditions that have previously been shown to be non-toxic, and future work should focus on maintaining this important constraint.

## 3. Gold Nanoparticles as Radiosensitizers

The use of high atomic number (Z) material to enhance radiation dose has been studied for more than 50 years. The interest in using high-Z material stems from the production of secondary electrons, such as photoelectrons, Auger electrons, and Compton electrons. These secondary products are effective at damaging DNA as well as ionizing surrounding water molecules, forming free radicals [110]. While the atomic number of tissue is approximately Z~7.5, materials with a higher atomic number used in the past such as Iodine (Z = 53) and gold (Z = 79) have a larger cross-section for absorption of radiation. For example, it was demonstrated in vitro that incorporating iodine into cellular DNA using iododeoxyuridine enhanced radiosensitivity at keV ranges by a factor of three [111]. The outcome of the in vitro study was also seen in an in vivo study, where an intratumoral injection of iodine and 200 kVp X-ray radiation suppressed the tumor growth by 80% [112]. In addition to having a great difference in mass attenuation between gold and soft tissue, gold has been shown to be biocompatible, simple, and economical to manufacture in many different shapes and sizes [113].

**Figure 5:**
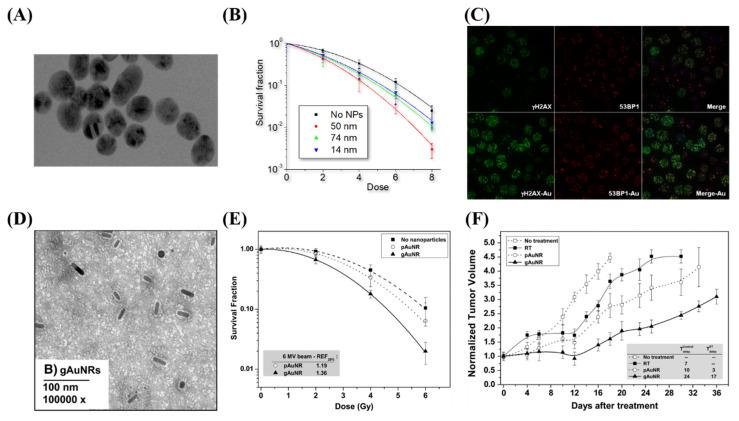
**Radiosensitization due to gold nanoparticles.** (**A**–**C**) Spheroidal GNPs improve radiosensitization in vitro, with the largest effect occurring with 50 nm GNPs, as they have the optimum uptake. This can be seen both through clonogenic assays as well as through imaging of double strand break foci with confocal microscopy. (**D**–**F**) GNRs displayed increased radiosensitization when targeted towards prostate cancer cell lines both in vitro through a clonogenic assay as well as in vivo through tumor volume measurements in a mouse model. Reproduced with permission from [117]. Copyright Elsevier, 2015.

Radiation dose enhancement due to GNPs was first demonstrated using 1.9 nm GNPs in a mouse model, in one of the pioneering studies in GNP-mediated radiation dose enhancement by Hainfeld et al. [29]. A radiation dose of 30 Gy with 250 kVp X-rays to subcutaneous tumors in mice resulted in a significant decrease in tumor volume. However, the concentration of gold in this study was considerably high, at 2.7 g Au/kg body weight, which is not clinically feasible. Furthermore, the use of kV energies, while allowing for prominent photoelectric absorption in gold, is hindered due to the reduced penetration for deep-set tumors. Thus, as previously discussed, optimization of the internalization of the GNPs into the tumor cells, both in-vitro and in-vivo, is required for ideal efficacy. Whenever gold was internalized in vitro, radiosensitization was achievable at MV energy ranges, at concentrations as low as 1 ng/g [25,114,115,116]. This was demonstrated by Chithrani et al. in Figure 5A–C, which found a 17% increase in radiosensitization at 6 MV with 50 nm spherical GNPs [25]. When moving to an in vivo environment, radiosensitization was seen at a delivered dose of 10 μg/g of body weight [117]. This was accomplished by Wolfe et al. using targeted GNRs, as seen in Figure 5D–F, where there was a 36% increase in radiosensitization in vitro in PC3 cells, and a significantly enhanced tumor-growth delay when treated in vivo [117]. The treated dose is a ~$1\times 10^{6}$ improvement over the original treatment seen in Hainfeld’s pioneering study. The addition of targeting and improvements in the optimization of uptake has allowed significant progress in facilitating the progress of gold nanomaterials to the clinic.

**Figure 6:**
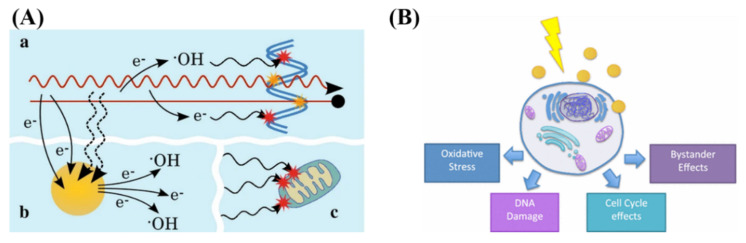
**Radiosensitization and radiobiological effects.** (**A**) Schematic showing chemical mechanism of GNP radiosensitization. While the radiation causes direct and indirect damage (yellow and red stars, respectively), there can be induction of secondary electrons and reactive oxygen species through gold nanoparticles. This can lead to damage to the DNA as well as secondary parts of the cell, such as the mitochondria. (**B**) GNPs can influence the cell through generation of reactive oxygen species, DNA damage, as well as cell cycle and bystander effects. Reproduced with permission from [110,118]. Copyright Springer Nature, 2016, 2017.

GNPs localized intracellularly increases the probability of ionization events leading to local enhanced deposition of energy causing more damage to tumor cells [25]. The physical mechanism of GNP radiosensitization, seen in Figure 6A, occurs within the first nanoseconds of exposure, and is based on the difference in mass energy absorption coefficients between gold and soft tissue, enabling dose enhancement. The range of electrons released from GNPs is short, only a few micrometers, causing highly localized ionizing events. Thus, to achieve any enhancement from GNPs in radiation therapy, GNPs must be delivered and internalized specifically by tumor cells.

The chemical mechanism of GNP radiosensitization occurs through the radiochemical sensitization of DNA by increasing catalytic surface activity and increasing radical generation from the GNP surface [60]. Despite the prevailing notion that GNPs are chemically inert, there is a growing body of evidence that, due to the electronically active surface of GNPs, they are capable of catalyzing chemical reactions [119]. Catalysis by GNPs occurs mainly through surface interaction with molecular oxygen, generating free radicals [60]. This seems more evident in small GNPs (<5 nm in diameter) where surface to volume ratio is greater [120]. When combined with irradiation, the catalytic effects appear to be enhanced, with smaller GNPs with larger surface areas yielding more ROS [121]. However, it has been shown that at all energy levels, the dose enhancement observed cannot be simply explained by physical or chemical mechanisms [122]. To explain this, a radiobiological effect must be occurring.

The main radiobiological mechanisms involved in the cell’s response to irradiated GNPs results are the production of reactive oxygen species (ROS), oxidative stress, DNA damage induction, potential bystander effects, and cell cycle effects, as explained by Rosa et al. in Figure 6B [118]. Oxidative stress can cause cellular damage to the cell, including the oxidation of lipids, proteins, and DNA, which can result in apoptotic and necrotic cell death [123]. The mitochondria do appear to play a role, and the data indicate loss of function due to high intracellular ROS levels. It has been shown that the use of 1.4-nm triphenyl monosulfonate (TPPMS)-coated GNPs resulted in a loss of mitochondrial potential through elevated oxidative stress, resulting in necrotic cell death [122]. There have also been studies suggesting that GNPs may cause cell cycle disruptions and induce apoptosis. Radiosensitivity varies throughout the cell cycle with S phase being where a cell is most radioresistant and G2/M phase being most sensitive [124]. This could also depend on cell type, expression of cyclin kinases, and NP characteristics such as coating and size. For example, the use of 1.9 nm GNPs in DU-145 and MDA-MB-231 resulted in an increase in sub-G1 population in DU-145 population but not in MDA-MB-231 [125].

The biocompatibility of GNPs has already been tested in a phase I clinical trial. Furthermore, both in vitro and in vivo studies have shown the possibility of using GNPs as a radiosensitizer at clinically feasible concentrations, as discussed previously. Radiotherapy can also be combined with chemotherapy (chemoradiation) in cases where the tumor is not localized anymore, but metastasized as well, or to reduce potential micro-metastases spread. We will discuss the recent research conducted towards adding GNPs to this chemoradiation protocol in the next section.

## 4. Rationale for Gold Nanoparticles in Chemoradiotherapy

Radiotherapy is mainly used to control the tumor locally as discussed previously. Chemotherapy is used to control the tumor metastasis. Therefore, a combination of chemotherapy and radiotherapy (chemoradiation) is being practiced in the clinic to treat patients with locally advanced disease. Considering the variety of drugs available for cancer treatment, the possible choice of sequencing of combined chemotherapy and radiation therapy is countless, and the treatment plan differs between each patient. The standard treatment sequence refers to chemotherapy regimen before a traditional external beam radiation therapy treatment [53]. Chemotherapy used prior to irradiation is expected to cause maximal tumor regression for locally advanced tumors. The major limitation of combining chemotherapy and radiation therapy is normal tissue toxicity, since either modality can cause major normal tissue toxicity [8]. The main problems currently associated with chemotherapy are the biodistribution of pharmaceuticals, the lack of drug-specific affinity towards the tumor, limited plasma half-life, poor solubility and stability in physiological fluids, and nonspecific toxicity [126]. GNPs, due to their high surface area-to-volume ratio, as well as a large number of surface bio conjugation possibilities, are an ideal platform for delivering pharmaceutics for chemotherapy [46,127,128,129]. The use of GNPs as drug delivery system (DDS) can improve the pharmacokinetics, the pharmacodynamics, and the biodistribution of various drugs, as well as allow for improved targeting to reduce normal tissue toxicity. Beyond being an effective radiosensitizer, GNPs allow for a 100- to 1000-fold increase in ligand density compared to that of liposomal or polymeric DDSs [55]. Thus, the combination of the GNPs with radiotherapy and chemotherapy is part of the natural progression of the exploration of GNPs as a complete treatment modality.

The conjugation of moieties such as chemotherapeutic agents to the surface of the GNPs can be done using various techniques. The most common method is through the use of thiol group-containing biomolecules [130]. The use of thiolated biomolecules allows for functionalization of the GNPs with various agents, such as DNA, peptides, antibodies, and proteins [131,132]. This is a very robust method, as the majority of anticancer drugs can be thiolated, so as to be compatible with GNPs as a DDS [133,134]. Furthermore, capping agents, such as carboxyl terminated PEG molecules, with a thiol bond can allow for further functionalization techniques [131,135]. A general overview of various drug loading techniques using GNPs was explored by Fratoddi et al. [136]. GNPs, due to their favorable surface chemistry, are a suitable drug carrier for use in chemotherapeutics, and may be available for use in a wide range of drug delivery applications.

**Figure 7:**
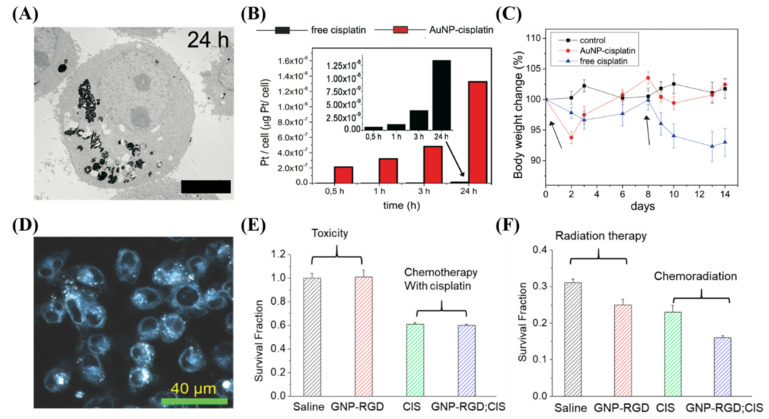
**Cisplatin and gold nanoparticles**. (**A**–**C**) Cisplatin conjugated GNPs lead to an increased deposition of platinum into A549 cells compared to the free drug in vitro, which led to less side effects in vivo with similar efficacy. Scale bar is 4 μm. (**D**–**F**) The use of free cisplatin to synergize with GNPs in a chemoradiation modality in vitro lead to a synergistic effect. Scale bar is 40 μm. Reproduced with permission from [116,147]. Copyright Public Library of Science, 2012; Copyright Multidisciplinary Digital Publishing Institute, 2018.

GNPs have been conjugated to a large variety of cytotoxic, anticancer drugs, and combined with radiation for improved efficacy. This includes paclitaxel, methotrexate, gemcitabine, doxorubicin, docetaxel, bleomycin, and platinum-based drugs like cisplatin [133,137,138,139,140,141]. Many different drugs can be used for different purposes, a few of which we will expand on. For instance, the antitumor activity of cisplatin was first discovered by Rosenberg and co-workers in 1960s, when they were examining whether electrical currents affect cellular division [142]. The researchers discovered that the inhibition of cellular division observed in the study was not due to electrical current, but platinum hydrolysis products formed from platinum electrodes. They reported that *cis*-tetrachlorodiammineplatinum (IV), *cis*-[PtCl_4_ (NH3)_2_], was the potent agent responsible for the inhibition. Cisplatin is now used to treat various types of cancers (i.e., cervical cancer, non-small-cell lung cancer, ovarian cancer, germ cell tumors, osteosarcomas, etc.), with a cure rate as high as 90% in testicular cancer [143]. However, long term cisplatin usage results in drug resistance [144]. To counteract this resistance, very high systemic doses of cisplatin should be administered, which results in severe systemic toxicity and poor patient compliance, limiting its clinical use [144,145,146]. It was shown recently that GNPs can be used to enhance damage caused by platinum-based anticancer drugs [147,148]. Comenge et al. conjugated cisplatin to GNPs and tested the efficacy of this DDS compared to the free drug along, as shown in Figure 7A–C [147]. They found that the use of GNPs led to 300 times more platinum being encapsulated in A549 cells in vitro, and while moving to in vivo, found similar efficacy but largely absent normal tissue toxicity. Yang et al. instead used free cisplatin along with GNPs in a combined chemoradiotherapy modality in vitro, seen in Figure 7D–F [116]. An additive relationship was discovered when treated with GNPs, cisplatin, and radiation in MDA-MB-231 cells. The use of GNPs may be an important avenue to explore when integrating cisplatin into chemoradiation protocols.

**Figure 8:**
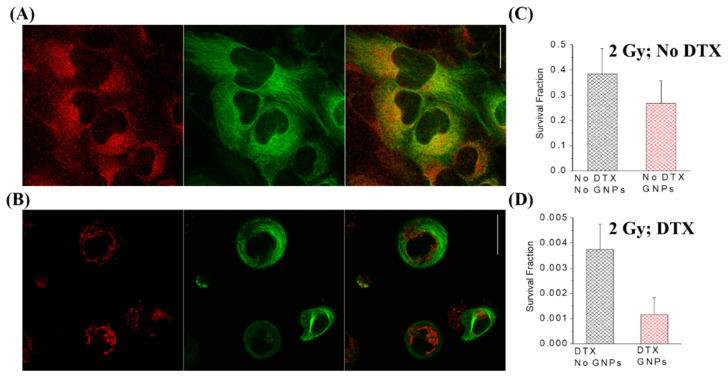
**Docetaxel and gold nanoparticles.** (**A**) Confocal imaging of GNPs (labelled in red) and the microtubule (MT) structure (labelled in green) in HeLa cells. (**B**) Confocal imaging of GNPs and MTs after treatment with 50 nM of DTX. (**C**) Radiosensitization of GNPs without DTX. (**D**) Radiosensitization of GNPs with 50 nM DTX, showing a synergistic effect. Scale bar is 25 μm. Reproduced with permission from [160]. Copyright British Journal of Radiology, 2020.

Another chemotherapy agent that is limited due to high normal tissue toxicity is docetaxel (DTX). DTX is a cytotoxic member of the taxanes and is an effective antimicrotubular agent that is effective in the treatment of multiple different types of cancers including head and neck, breast, prostate, and non-small-cell lung cancer [149,150,151,152]. Docetaxel’s mechanism of action is primarily through the ability to enhance microtubule assembly and stabilize free microtubules within the cytoplasm, thus preventing their depolymerization during normal cell division [153]. This has many consequences for the fate of the cell, including inhibition of progression through the cell cycle and inevitably death via mitotic catastrophe, depending on the dose [154]. Francois et al. tested DTX-conjugated GNPs on MCF7 and HCT15 cells and found a 2.5 times more efficient response compared to free DTX [139]. DTX has also been shown to block the cell cycle at the G_2_/M phase [155]. This is critical because the G_2_/M phase has shown special sensitivity to the ionizing radiation, causing more cell death [156]. Moreover, by arresting tumor cells in the M phase of the cell cycle, it synergizes the lethal effects of radiotherapy, thereby serving as an ideal radiosensitizer. Both in vitro and in vivo studies have demonstrated the synergistic effects of DTX when combined with radiotherapy [157,158]. Furthermore, it has been shown that the uptake of NPs, including GNPs, is increased when the cell population is synchronized in the G_2_/M phase [85,159]. This suggests the use of DTX concomitantly with other drugs or radiation, which was tested by Bannister et al. as seen in Figure 8 [160]. DTX used as a synchronizing agent when paired with GNPs lead to higher uptake, a higher localization of the GNPs to the nucleus, and a larger, synergistic response to radiotherapy.

**Figure 9:**
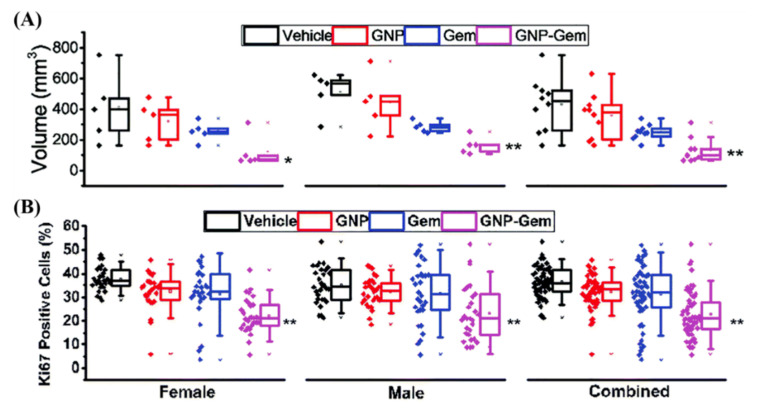
**Gold nanoparticle mediated delivery of Gemcitabine.** (**A**) Gemcitabine-conjugated GNPs showed a significant increase in efficacy when treating mice, as measured through volume. (**B**) KI67, a marker for proliferation, also showed reduced proliferative cells when treated with the GNP complex compared to the free drug alone, signifying improved efficacy. Black = vehicle, Red = GNP, Blue = Gem and Purple = GNP-Gem. * and ** denote *p* < 0.05 and *p* < 0.01 compared to vehicle-treated group respectively. Reproduced with permission from [169]. Copyright Royal Society of Chemistry, 2017.

Normal tissue toxicity is an issue in escalating current dose regimes in many tumors. However, in pancreatic cancer, despite advancements in chemotherapy and radiotherapy, the current 5 year survival rate is only 9% [161]. Gemcitabine is a pyrimidine analog that is a mainstay treatment of adenocarcinoma of the pancreas, but is also used for treatment of bladder and non-small cell lung cancer [162,163,164]. Upon cellular encapsulation, gemcitabine is phosphorylated to its active diphosphate and triphosphate metabolites, which inhibit RR and DNA synthesis, respectively [165]. Despite its prominent use in the clinic, only a small portion of the drug is converted to its active forms. Up to 90% of the injected dose is collected from urine one week after treatment, with 75% of that being in the first 24 h [166]. The use of gemcitabine-conjugated GNPs could be an avenue for both improved uptake of the drug as well as improved efficacy in the very deadly pancreatic disease. It has been shown that 20 nm GNPs by themselves can sensitive pancreatic cell to the effects of gemcitabine by Huai et al. [167]. This is explained by showing that GNPs inhibited epithelial to mesenchymal transition and reduced cancer cell stemness—possible causes of anticancer drug resistance [168]. Furthermore, Pal et al. showed in Figure 9 that gemcitabine-conjugated GNPs that specifically target pancreatic cancer cells with a plectin-1 peptide have improved efficacy in situ compared to the free drug alone [169]. The use of gemcitabine with radiotherapy has also been shown to be more effective than the drug alone through clinical trials [170]. Thus, in the future, the addition of gemcitabine-conjugated GNPs to a radiotherapy protocol may prove highly beneficial.

The use of GNPs as chemotherapeutic DDSs is increasing, and the combination of chemotherapy with radiotherapy remains one of the most effective treatment modalities available in the clinic. A brief summary of recent studies, no older than 2016, involving the use of GNPs with chemotherapy and radiotherapy, can be seen in Table 1. The use of GNPs in combination with anticancer drugs and radiation is still limited in literature; however, the published work thus far shows a trend of improved dose response to the tumor coupled with reduced normal tissue toxicity. Further studies need to be completed, however, before translation to the clinic.

## 5. Future Considerations

A large issue that is plaguing nanotechnology in general is that a very low (~0.7%) portion of the administered dose is being delivered to the tumor [177]. While Wilhelm et al. describe the issue as improving understanding of the processes present in the body that inhibit the uptake of NPs, and then optimizing those processes, a more personalized approach could be introduced. Personalized medicine involves the analysis of a patient’s genetic code in order to have a better understanding of the potential response to treatment [178]. This is a very important avenue to explore, as it has been estimated that any class of anticancer drug used is ineffective in 75% of patients [179]. This is a result of no two cancers from different patients being the same. Beyond using genomic information to improve each individual’s cancer treatments, the use of an in vitro model that can better mimic the in vivo environment present in a patient may allow for the actual testing of treatment prior to administration. This can be achieved through the use of three-dimensional organoid models [180].

**Figure 10:**
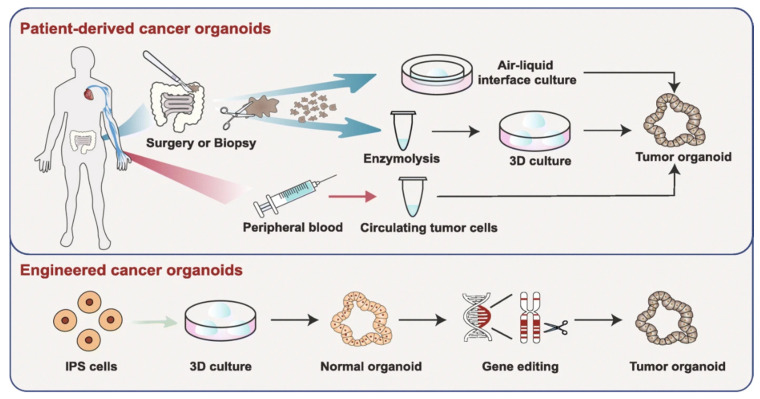
Organoid models toward personalized medicine. Patient-derived cancer organoids can be derived from surgically resected/biopsied tissues and circulating tumor cells. Furthermore, using gene-editing, normal spheroids can be mutated into tumor organoids. Reproduced with permission from [180]. Copyright Springer Nature, 2019.

Organoid models have many advantages if implemented into a personalized medicine protocol. The use of a patient’s own cells, as described in Figure 10 by Fan et al., allows for the maintenance of the heterogeneity present [181]. Furthermore, normal spheroids can be engineered to have similar genetic alterations present in a patient, as discovered using their genomic information, through the use of gene-editing [182]. There are many other advantages as well, including low-cost generation, and quick (~4 weeks) results can be obtained. The capability of organoids to enable drug screening in an in vitro environment is being widely explored [180]. Furthermore, the use of organoid models has recently been tested on a chemoradiotherapy treatment of advanced rectal cancer and was able to accurately predict the response [183]. In the future, the use of organoids to screen chemoradiotherapy protocols with GNPs may enable an accurate assessment of response and allow for tailored, personalized medical care.

Spheroids, and patient derived organoids, should be seen as avascular tumors, with limitations. To move towards personalized medical care with GNPs—using organoids as an assessment tool—certain strategies will have to be employed and obstacles overcome [184]. First, many more preclinical studies will have to be undertaken involving the use of GNPs with spheroids and their ability to accurately predict tumor response. This will have to be a large expanse of research, with many different types of treatment options including chemotherapy and radiotherapy as well as combined modalities. Second, scalability is very important: a high throughput method of testing efficacy of drugs and radiation modalities will have to be elucidated, as well as producing the organoids in a large-scale manner. Work is under way to improve production of spheroids as well as protocols for high throughput drug and radiation testing [185,186,187]. However, translation to the clinic will require more work at the bio–nano interface.

The use of GNPs in this workflow has limited published work, with most research focusing on individual treatment modalities, and not overall high throughput methods for translation to personalized medicine. Towards this, cheap and efficient GNP systems that can be easily functionalized with various moieties such as anticancer drugs or targeting ligands need to be designed for mass-scale production. Furthermore, comparisons of GNP-treated spheroids and organoids with in vivo models must be undertaken for improved confidence for translation to the clinic. Finally, it must be accepted the spheroids are a simplistic model when compared to an in vivo environment and will not be able to predict everything. However, despite this, the use of GNPs with organoid models for personalized medicine may be able to help save lives and improve the quality of lives in the future.

## 6. Conclusions

In the pursuit of improved cancer therapeutics, the use of GNPs offers the potential of improving on many different facets of the treatment process. Despite progress, the translation of GNPs to clinical practice has been limited due to the lack of coordination between researchers and clinicians. Many advances covered in this review aim to address issues that have arisen in the past, including targeted therapy, and the combination of radiotherapy and chemotherapy paired with GNPs for improved efficacy. However, it is still important to improve upon the current research so that translation to the clinic can be expedited.

## Figures and Tables

**Table 1:** Recent applications of anticancer drugs with gold nanoparticles in drug delivery and combined radiation therapy with clinically relevant energies.

Nanoparticle Complex	Treatment Parameters	Modality	Experimental Outcomes	Cell Line/Tumor Model	Ref.
PTX-TNFα-PEG-GNPs	32.6 nm GNPs; 2.5 mg/kg dose	Chemotherapy	Selective delivery of nanoparticles to tumor and improved efficacy	Ovarian Cancer Cell Line (A2780); B16/F10 tumor-bearing C57BL/6 mice	[133]
DOX-PEG-GNPs	41 nm GNPs; 6 mg/kg dose	Chemotherapy	Dramatically reduced normal tissue toxicity	Ovarian Cancer Cell Line (A2780); CD-1 mice	[138]
BLM-DOX-PEG-GNPs	13 nm GNPs; 10–100 nM dose	Chemotherapy	Cancer cell environment-mediated drug release and improve EC50	Cervical Cancer Cell Line (HeLa)	[140]
CIS-GLC-PEG-GNP	20 nm GNPs; 10 mg/kg dose; 25 Gy at 6 MV	Chemo-radiotherapy	Similar effect to free cisplatin; dramatically improve result when combined with radiation	Skin Cancer Cell line (A-431); A-431 tumor-bearing mice	[171]
DOX@GNPs	2 nm GNPs; 5 mg/kg dose	Chemotherapy	Efficient renal clearance with effective targeting. Reduced normal tissue toxicity with improved antitumor efficacy	Breast Cancer Cell lines (MCF-7 and MDA-MB-231); Murine Mammary 4T1; CD-1 Mice	[172]
PDC-PEG-GNPs	25–50 nm GNPs; 0–50 µM dose	Chemotherapy	Improved half-life of drug, similar cytotoxicity towards target cells, and active for longer	Murine Lymphoma cells (A20)	[173]
Alginate co-loaded with GNPs and CIS	44 nm NP; 20 µg/mL dose of GNP with 5 µg/mL CIS; 4 Gy at 6 MV	Chemo-radiotherapy with photothermal therapy	ACA and radiotherapy saw improved efficacy over cisplatin and radiation. The addition of photothermal therapy further improved therapeutic results.	Cervical Cancer Cell line (KB)	[174]
5-FU/GSH-GNPs	9–17 nm GNPs; 0.5–1.5 mg/mL dose	Chemotherapy	Better anticancer effect against the cancer, and reduced drug doses as a result	Colorectal Cancer Cell lines isolated from patients	[175]
CS-GNPS-DOX	21 nm GNPs; 0.05–0.3 mM dose; 0.5, 1, and 3 Gy at 6 MV	Chemo-radiotherapy	Enhanced treatment results including lowered survival fraction, increased apoptosis, and increased DNA damage	Breast Cancer Cell line (MCF-7)	[176]
GNP-PEG-RGD; CIS	10 nm GNPs with 435 nM CIS; 0.3 nM dose; 2 Gy at 6 MV	Chemo-radiotherapy	Improved efficacy of treatment compared to cisplatin and radiation alone	Breast Cancer Cell line (MDA-MB-231)	[116]
GNP-PEG-RGD; DTX	17.2 nm GNPs; 0.2 nM GNPs with 50 nM DTX; 2 Gy at 6 MV	Chemo-radiotherapy	Improved retention of GNPs due to cell synchronicity induced by DTX. Synergistic therapeutic effect found when GNPs and DTX combined	Breast Cancer Cell line (MDA-MB-231) and Cervical Cancer Cell line (HeLa)	[160]

GNP: Gold Nanoparticle; PAC: Paclitaxel; TNF: Tumor Necrosis Factor; PEG: Polyethylene Glycol; DOX: Doxorubicin; BLM: Bleomycin; CIS: Cisplatin; GLC: Glucose; PDC: Peptide-drug-conjugate containing chlorambucil, melphalan, or bendamustine; 5-FU: 5-Fluorouracil; GSH: Glutathione; CS: Chitosan; RGD: arginyl-glycyl-aspartic acid tripeptide; DTX: Docetaxel
